# Phytoplankton community and algal toxicity at a recurring bloom in Sullivan Bay, Kabetogama Lake, Minnesota, USA

**DOI:** 10.1038/s41598-019-52639-y

**Published:** 2019-11-06

**Authors:** Victoria G. Christensen, Ryan P. Maki, Erin A. Stelzer, Jack E. Norland, Eakalak Khan

**Affiliations:** 1 U.S. Geological Survey, Upper Midwest Water Science Center, 2280 Woodale Drive, Mounds View, MN 55112 USA; 2 North Dakota State University Environmental and Conservation Sciences Program, Fargo, ND 58102 USA; 3 Voyageurs National Park, 360 Highway 11 East, International Falls, MN 56649 USA; 4 U.S. Geological Survey Ohio Water Microbiology Laboratory, 6460 Busch Blvd STE 100, Columbus, OH USA; 5 North Dakota State University, Morrill Hall - Room 205A, 1230 Albrecht Blvd, Fargo, ND 58102 USA; 6 University of Nevada, Las Vegas, SEB 3134, 4505S Maryland Pkwy, Las Vegas, NV 89154 USA

**Keywords:** Water microbiology, Limnology

## Abstract

Kabetogama Lake in Voyageurs National Park, Minnesota, USA suffers from recurring late summer algal blooms that often contain toxin-producing cyanobacteria. Previous research identified the toxin microcystin in blooms, but we wanted to better understand how the algal and cyanobacterial community changed throughout an open water season and how changes in community structure were related to toxin production. Therefore, we sampled one recurring bloom location throughout the entire open water season. The uniqueness of this study is the absence of urban and agricultural nutrient sources, the remote location, and the collection of samples before any visible blooms were present. Through quantitative polymerase chain reaction (qPCR), we discovered that toxin-forming cyanobacteria were present before visible blooms and toxins not previously detected in this region (anatoxin-a and saxitoxin) were present, indicating that sampling for additional toxins and sampling earlier in the season may be necessary to assess ecosystems and human health risk.

## Introduction

Cyanobacteria, often referred to as blue-green algae, are the oldest oxygen-producing organisms^1^ and have had major effects on the Earth^2^. They provide food for fish and are important for multiple trophic levels but can be harmful to ecosystems by contributing to hypoxia, disrupting the food web, and releasing toxins.

Specific cyanobacteria have traits that allow them an advantage over other prokaryotic cyanobacteria and eukaryotic algae, and responses to environmental conditions can be taxon-specific^3^. For example, *Anabaena* can fix nitrogen from the atmosphere^4^ and are adapted to relatively high light environments and conditions. Conversely, *Aphanizomenon* are adapted to relatively low light conditions^5^, and *Planktothrix* can withstand continuous mixing in shallow lakes^6^. Additionally, cyanobacteria size and density can affect buoyancy^7^. *Microcystis*, for example, accumulates carbohydrates to regulate density as a type of “ballast” mechanism^7^. These competitive advantages are a key to understanding dominant forms of phytoplankton during the formation of blooms—the visible and rapid growth of cyanobacteria that typically occurs at the water surface.

The toxins in these cyanobacterial blooms (cyanotoxins) can adversely affect humans, animals, and ecosystems. Whereas short-term human health responses to cyanobacterial toxin exposure are known^8,9^, long-term effects of human or animal exposure, as well as ecosystem effects are largely unknown. This gap in our scientific knowledge is a concern in areas with recurring blooms. Moreover, geographical distribution of cyanotoxin occurrence from existing literature indicates a substantial data gap in a large expanse of the north central USA^10^. Therefore, we studied the temporal variability of the phytoplankton community and the relation to cyanotoxins at a remote recurring bloom site in Sullivan Bay, Kabetogama Lake, Voyageurs National Park (VOYA).

Many conditions in combination, such as nutrients from human activities^2^, agricultural practices^11^, or lower salinity levels^12^, can set up a water body to be susceptible to bloom formation. However, with the exception of internal loading at various locations in Kabetogama Lake^13^ and the extensive clay soil deposits on the lake’s south side^14^ that could account for high concentrations of nutrients in the lake, many of the nutrient sources common to lakes with frequent algal blooms are absent from VOYA due to its remote location. Therefore, this study provided a unique opportunity to evaluate seasonal changes in phytoplankton composition as it relates to toxin production in an area without the substantial urban and agricultural nutrient sources and in an area without significant cyanotoxin occurrence reported in peer reviewed literature^10^. The results may be useful to similar remote or northern temperate lakes experiencing annual algal blooms.

## Background

Cyanobacterial algal blooms in Kabetogama Lake, one of more than 30 lakes in VOYA, have frequently produced the toxin microcystin^15^. During a 2008–09 study at VOYA, microcystin (total microcystin and nodularin expressed as relative amount of microcystin-LR) was reported in 11 of 14 bloom samples (78%)^13^, and 7 of 14 bloom samples (50%) exceeded the 1-μg/L guideline for drinking water established by the World Health Organization (WHO)^16^. Two samples exceeded 20 μg/L, putting them into the WHO high-risk category for recreational exposure^17^. Moreover, VOYA lacked data for other algal toxins, such as anatoxin-a and saxitoxin. The WHO recommends changes in monitoring for cyanobacterial blooms with microcystin-LR concentrations that exceed 10 μg/L. The WHO also recommends a moderate health alert when concentrations exceed 20 μg/L in recreational water^17^.

Substantial biovolumes of two cyanobacteria known to produce toxins, *Dolichospermum* and *Microcystis*, have composed much of the phytoplankton community in Kabetogama Lake blooms^15^. *Dolichospermum, Microcystis*, and *Planktothrix* are all commonly known to produce microcystins^18^, whereas anatoxin-a and saxitoxin are known to be produced by additional species, such as *Aphanizomenon*, *Lyngbya*, or *Cylindrospermopsis*^17,19^. The pelagic form of *Anabaena* has recently been identified as *Dolichospermum*^20^ and therefore for our study, we refer to occurrences of this genera as *Dolichospermum*.

Production of microcystin can only occur if the microcystin synthetase (*mcy*) gene is present in the genome^21^. Individual strains within a microcystin-producing genus may include both the toxic strains and nontoxic strains, in other words with or without the *mcy* genes, respectively^18^. These toxic and nontoxic strains can be differentiated in about 3 hours by quantitative polymerase chain reaction (qPCR), a molecular detection method^22,23^.

The purpose of this research was to better understand changes throughout the open water season in cyanobacterial community structure (in terms of abundance, species composition, and toxin production capability). Specific objectives of this paper are to (1) identify and quantify toxin producing cyanobacteria with molecular tools (qPCR); and (2) determine the relations between toxin production and changes in phytoplankton community structure.

The novelty of this study is that it included the collection of samples after ice out occurred and before any algal blooms were visible, whereas most studies only collect these types of samples when algal blooms are visible. Additionally, we analyzed for anatoxin-a and saxitoxin, two toxins that understudied in freshwater and are covered by only 9% and 27% of published cyanotoxin papers, respectively^10^, giving us more understanding on these toxins. The knowledge gained from this study may be valuable to other researchers and resource managers, who want to better predict, manage, and mitigate the occurrence of toxic cyanobacterial blooms.

## Site Description

Kabetogama Lake lies within VOYA, but about 20 km of private shoreline surrounds the south end of the lake^14^. Kabetogama Lake is 10,425 ha, with a watershed to lake ratio of 196.7, a maximum depth of 24.3 m, and a mean depth of 9.1 m^14^. Land use in the Kabetogama Lake watershed is primarily forested (59%), with water features making up another 38%, and grasslands 2%^14^. Small pockets of developed areas and agriculture, in the form of hay fields, and make up less than 1% of the watershed. Kabetogama Lake suffers from annual algal blooms unlike most of VOYA lakes, which are primarily oligotrophic to mesotrophic. Kabetogama Lake is noticeably different than the other large lakes of the park in that thermal stratification occurs infrequently, whereas thermoclines are typically present throughout the summer in the other large lakes. However, dissolved oxygen concentrations fell below accepted standards in Kabetogama Lake when thermoclines developed during hot, still weather^24^.

Kabetogama Lake also has different water chemistry than the park’s other large lakes^14^, and differences in water quality occur between the shoreline, mid-lake, and individual bays^15^. Additionally, Kabetogama Lake has higher specific conductance, nutrient, and chlorophyll-*a* concentrations^14^ and is shallower than other park lakes, resulting in higher temperatures, which have been connected with algal blooms in other systems^25^. Moreover, at least one study points to the importance of local environmental conditions in relation to microcystin production^26^.

Sullivan Bay within Kabetogama Lake is near the southern border of the park, with depths ranging from 1.8 to 4.9 m^27^. Sullivan Bay is characterized by higher dissolved solids and alkalinity than the rest of the lake, reflecting inflow from Ash River^27^. A recurring bloom site in Sullivan Bay (U.S. Geological Survey site number 482542092493701) was selected for intensive algal bloom sampling and a comparison to phytoplankton species. Sullivan Bay is shallow, polymictic^14,27^, and a popular recreational location with cottages and resorts upstream. The degraded water quality in this bay has historically been attributed to the inflow from Ash River and its rich geological substrates^14^, but no recent source allocation has been conducted.

## Results

### Seasonal changes in phytoplankton community

We collected 12 samples at the Sullivan Bay algal bloom site (482542092493701) and analyzed these samples for phytoplankton abundance and community structure. Detailed taxonomic identification and enumeration data from these samples were published in the U.S. Geological Survey (USGS) ScienceBase Catalog^28^.

Total cell abundance and total biovolume (Table 1) varied early in the season but had two distinct peaks (August 1, 2016, and August 16, 2016). Cell abundance is the total number of cyanobacterial or algal cells in the sample per unit volume; biovolume indicates algal mass by volume^23^. In other words, cell abundance and cell biovolume are different because some algal cells are larger than others and therefore one cell may have a greater biovolume than others. The summer peaks are followed by a rapid decline that continued through Sept. 13, 2016.

**Table 1:** Summary of phytoplankton abundance and biovolume in water samples from Kabetogama Lake, Sullivan Bay, Northwest, near Ash River (U.S. Geological Survey Site No. 482542092493701), Voyageurs National Park, MN, USA, 2016.

Date	Abundance (cell/L)	Total biovolume (μm^3^/L)	Cyanobacteria biovolume percent
6/21/2016	26,156,702	8,087,275,833	7.0
6/27/2016	63,105,589	7,026,359,688	24
7/6/2016	40,101,966	2,554,264,945	30
7/12/2016	85,944,746	8,749,585,308	39
7/19/2016	119,889,980	5,836,783,205	43
7/26/2016	229,360,060	18,597,701,465	53
8/1/2016	286,569,740	19,499,667,005	53
8/10/2016	156,776,705	10,854,356,404	60
8/16/2016	289,829,158	25,872,678,424	67
8/23/2016	282,470,651	11,604,888,019	69
8/30/2016	264,377,500	9,544,215,996	67
9/13/2016	34,668,911	2,050,802,261	12

[cell/L, phytoplankton cells per liter of water; mm^3^/L, cubic micrometers of total mass per liter of water]

**Figure 1:**
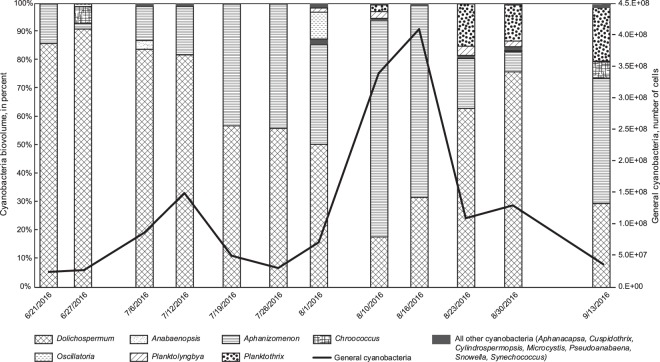
Cyanobacteria species as percent total biovolume compared to general cyanobacteria, of samples collected from Kabetogama Lake, Sullivan Bay, Northwest, near Ash River (U.S. Geological Survey site number 482542092493701), Voyageurs National Park, MN, USA, 2016.

In general, the percent cyanobacterial biovolume ranged from 7% in June to nearly 70% in August (Table 1). Among the cyanobacteria recorded early in the season are *Dolichospermum*, *Aphanizomenon*, and *Chroococcus* (Fig. 1). In late June, *Aphanocapsa*, *Planktolyngbya*, *Planktothrix (Oscillatoria)*, and *Pseudanabaena* emerge in water samples^28^. Generally, *Dolichospermum* dominates through mid-July. However, from mid-July to mid-August, samples consist primarily of *Aphanizomenon*, a producer of anatoxin-a and saxitoxin, which peaked on August 16, 2016. In late August, *Planktothrix* and *Dolichospermum* dominated. Finally, in the sample collected in mid-September, *Aphanizomenon* dominated once again.

### Seasonal changes in cyanobacterial gene results

Both general cyanobacterial counts and genus specific counts generally show a seasonal pattern, with higher counts in late summer (Table 2). However, not all genera peaked at the same time. For example, general *Dolichospermum* counts were greatest on August 10. Following a rapid decline in *Dolichospermum*, *Planktothrix* counts were greatest about a week later, on August 16 (Table 2). Anatoxin-a toxin genes (*anaC*) and saxitoxin toxin genes (*sxtA*) both peaked on August 16, 2016 (Table 3) with a pattern similar to general cyanobacteria and general *Planktothrix* (Table 2).

**Table 2:** Results of general cyanobacterial gene analysis by quantitative polymerase chain reaction (qPCR) in water samples from Kabetogama Lake, Sullivan Bay, Northwest, near Ash River (U.S. Geological Survey Site No. 482542092493701), Voyageurs National Park, MN, USA, 2016.

Date	General qPCR (gene copies per 100 milliliters)			
	Cyanobacteria	*Microcystis*	*Planktothrix*	*Dolichospermum*
6/21/2016	24,000,000	<810	19,000	500,000,000
6/27/2016	27,000,000	5,000	12,000	1,100,000,000
7/6/2016	87,000,000	2,500	<540	1,400,000,000
7/12/2016	150,000,000	40,000	41,000	20,000,000,000
7/19/2016	50,000,000	38,000	250,000	1,600,000,000
7/26/2016	30,000,000	1,600	160,000	1,500,000,000
8/1/2016	71,000,000	24,000	1,200,000	5,300,000,000
8/10/2016	340,000,000	180,000	34,000,000	120,000,000,000
8/16/2016	410,000,000	210,000	97,000,000	3,500,000,000
8/23/2016	110,000,000	70,000	24,000,000	3,600,000,000
8/30/2016	130,000,000	5,800	41,000,000	2,000,000,000
9/13/2016	33,000,000	E820	11,000,000	55,000,000

[DNA, deoxyrhibonucleic acid; mcyE, cyanobacterial microcystin synthetase gene copies; <, less than or below the detection limit; E, estimated value indicates above the detection limit but below the quantification limit].

**Table 3:** Results of gene specific analysis by quantitative polymerase chain reaction (qPCR) in water samples from Kabetogama Lake Sullivan Bay, Northwest, near Ash River (U.S. Geological Survey Site No. 482542092493701), Voyageurs National Park, MN, USA, 2016.

Date	Genus specific DNA by qPCR (gene copies per 100 milliliters)				
	*Microcystis* mcyE	*Planktothrix* mcyE	*Dolichospermum* mcyE	Anatoxin-a (*anaC*)	Saxitoxin (*sxtA*)
6/21/2016	<390	E2,800	160,000	<960	E560
6/27/2016	E700	<33,000	410,000	2,100	2,800
7/6/2016	E750	47,000	520,000	3,800	1,600
7/12/2016	10,000	<33,000	740,000	5,200	10,000
7/19/2016	12,000	<33,000	640,000	4,500	41,000
7/26/2016	1,200	<33,000	230,000	4,200	14,000
8/1/2016	13,000	62,000	1,200,000	23,000	160,000
8/10/2016	79,000	1,400,000	1,500,000	43,000	1,100,000
8/16/2016	39,000	37,000,000	420,000	95,000	14,000,000
8/23/2016	13,000	3,200,000	530,000	2,800	440,000
8/30/2016	E640	28,000,000	160,000	1,800	42,000
9/13/2016	E400	3,700,000	<1,400	E1,300	E660

[DNA, deoxyribonucleic acid; mcyE, cyanobacterial microcystin synthetase gene copies; *anaC*, anatoxin-a toxin gene copies; *sxtA*, saxitoxin toxin gene copies; <, less than or below the detection limit; E, estimated value indicates above the detection limit but below the quantification limit].

### Toxin production

In addition to examining the potential for toxin production using the qPCR molecular method, samples were analyzed for the cyanotoxins anatoxin-a, microcystin, and saxitoxin by enzyme-linked immunosorbent assays (ELISA) to obtain concentrations.

**Figure 2:**
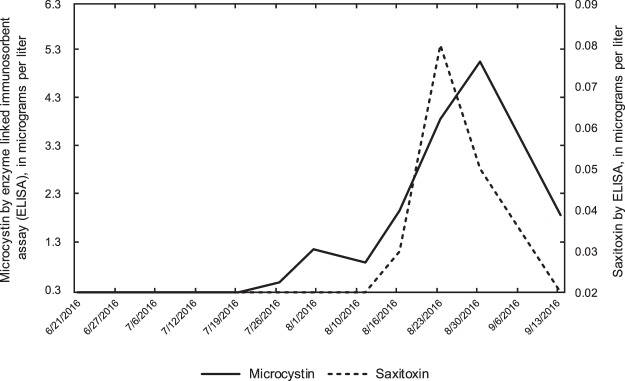
Graph of microcystin and saxitoxin concentrations determined with enzyme-linked immunosorbent assay in water samples from Sullivan Bay in Kabetogama Lake near Ash River, Minnesota (U.S. Geological Survey site number 482542092493701), 2016.

Toxin analysis by ELISA (Fig. 2), resulted in lower microcystin-LR concentrations than previous sampling at Kabetogama Lake^13^; however, previous samples were generally collected during active blooms. Microcystin-LR was detected in about 58% of samples (7 of 12). Anatoxin-a and saxitoxin detections were infrequent, with anatoxin present in 1 of 11 samples (0.31 μg/L on August 1, 2016) and saxitoxin present in 25% of samples (3 of 12 samples).

## Discussion

Cyanobacterial blooms tend to occur repeatedly in the same water supply^16^ and the long-term consequences of repeated exposure to cyanobacteria or cyanotoxins are unknown. This is an important issue locally, where a number of lakes have experienced cyanobacterial blooms resulting in dog deaths^29,30^, and regionally, where both dog deaths and human illness have been reported^31^. Microcystin has been well-documented throughout the region^19,29^; however, anatoxin-a and saxitoxin are understudied in freshwater, particularly in the north central USA^10^.

Temporal changes in the water column often affect phytoplankton dynamics^32^. For example, light and nutrient availability are often correlated with increased phytoplankton growth and temperature increases can result in a decrease in green algae^32^, which may open the door for cyanobacteria to dominate in late summer. Cyanobacterial species presence, in turn, determines which toxins can be produced; although not all strains of a particular species are capable of producing toxins^21^. In this study, total phytoplankton biovolume peaked prior to the peak in cyanobacterial biovolume percent (Table 1), which is consistent with an accepted pattern of phytoplankton succession in northern temperate lakes^33^. However, our study focused on the relation between community change throughout the open water season and toxin production (and/or presence of genes for toxin production) and we note an additional pattern of a genus specific *Microcystis* (*mcyE*) peak (August 10, 2016; Table 3) before the general *Microcystis* gene counts (August 16, 2016; Table 2). The *Microcystis* (*mcyE*) toxin gene counts in turn peaked prior to the peak in microcystin toxin concentrations (August 30, 2016; Fig. 2). These temporal differences provide insight into phytoplankton and cyanobacterial dominance throughout the open water season.

*Dolichospermum*, *Microcystis*, and *Planktothrix*, three toxin-producing genera, were prevalent during August; however, each genus also had a small peak in mid-July^28^. All three toxin producers were present in 10 of 12 (83%) samples collected in 2016 (Table 2), and whereas microcystin is known to recur in waterbodies, anatoxin-a occurrence is less consistent through time^22^. Both saxitoxin (*sxtA*) and anatoxin (*anaC*) genes also peaked during this period, prior to field observations of serious or extreme blooms at the surface. Although toxin concentrations were relatively low early in the season (Fig. 2) we cannot rule out the possibility of toxin presence early in the open water season before visible blooms are present. This reinforces the concept that one cannot simply look for the absence of a visible bloom as an indication of the absence of cyanotoxins because toxins may be present in the absence of a bloom and may have been produced by benthic taxa or by planktonic cyanobacteria that have since lysed. Conversely, the presence of a bloom does not always indicate the toxin is present, and high toxin levels can occur when *mcyE* genes are not present^34^.

Additionally, results of phytoplankton identification^28^ revealed the presence of *Aphanizomenon*, a known producer of anatoxin-a and saxitoxin. Because most toxin is produced in mid- to late August (Fig. 2), one might conclude that *Aphanizomenon* is responsible due to its abundance in mid-August (Fig. 1). However, because several possible toxin producing cyanobacterial species exist at once in the lake (*Dolichospermum*, *Microcystis*, *Planktothrix*, and *Aphanizomenon*), any of them has the potential to become dominant or form blooms in response to changed conditions^18^, which highlights the importance of studying the co-occurrence of multiple toxin producers. In addition, both toxic and non-toxic strains of cyanobacteria were present in blooms and it is possible that the main toxin producer is not the dominant genus in terms of cell abundance or biovolume.

Microscopic analysis of the samples also identified c.f. *Cylindrospermopsis* in one sample during the current study (August 1, 2016^28^). *Cylindrospermopsis*, which is generally found in tropical and subtropical lakes^16,35^, has been reported to produce the toxin cylindrospermopsin^36^. However, other researchers reported *Cylindrospermopsis* strains in the USA were genetically different from strains found in other parts of the world^37,38^ and lacked the capability to produce cylindrospermopsin^39^. We did not analyze for cylindrospermopsin in 2016, but 2017 qPCR gene assays and ELISA tests^40^ indicated its presence in samples from Kabetogama Lake.

The qPCR analysis helped us clarify when toxic and non-toxic strains occurred. Although cell count information may be used as a preliminary assessment of the hazard presented by the sample^17^, were we to rely on microscopic phytoplankton analysis alone to determine the cyanobacterial composition of the samples, several misconceptions are possible. Microscopic phytoplankton analysis without qPCR analysis cannot differentiate between toxic and non-toxic strains of these genera. Due to the lack of differentiation, we could mistakenly conclude that a bloom was capable of producing toxins when it was not, based on a microscopic analysis with high numbers of cyanobacteria present. Moreover, cyanobacteria cell abundance is often used as a surrogate for microcystin toxin values^41^, however, were we to compare cell abundance to microcystin concentrations, most samples from Kabetogama would be in the moderate risk category. The use of cell counts to estimate hazards can overestimate the risk to recreational users^42^. Therefore, the use of the qPCR technique for this study was valuable for differentiating whether the bloom was capable of producing a toxin.

**Figure 3:**
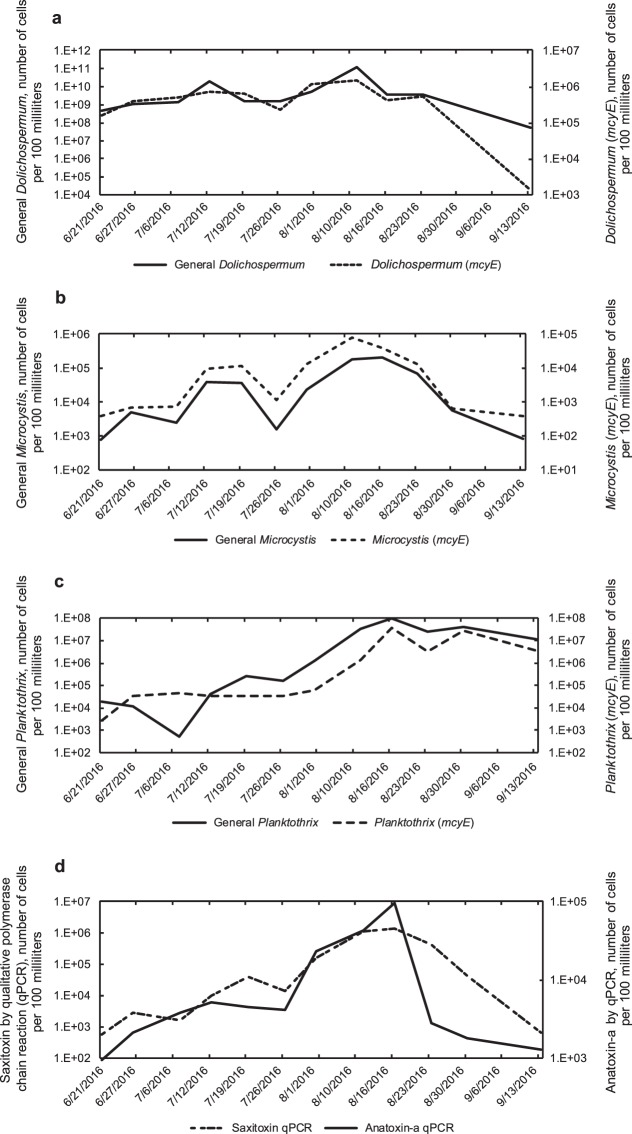
Graphs of (**a**) *Dolichospermum* and *Dolichospermum* (*mcyE*), (**b**) General *Microcystis* and *Microcystis* (*mcyE*), (**c**) general *Planktothrix* and *Planktothrix* (*mcyE*), and (**d**) anatoxin-a and saxitoxin determined with quantitative polymerase chain reaction, Kabetogama Lake in Sullivan Bay, near Ash River, Minnesota (site A1, U.S. Geological Survey site number 482542092493701), 2016.

When we compared algal biovolume data to general cyanobacterial gene abundance, which included toxic and non-toxic strains (Fig. 1), general cyanobacterial gene abundance peaked prior to the peak in algal biovolume (August 16 versus August 23). This may be an indication that smaller cyanobacteria are present during the earlier peak. *Dolichospermum* microcystin toxin genes (*mcyE*) had a slight peak (August 1, 2016; Fig. 3a) prior to the general *Dolichospermum* genes (August 10; Fig. 3a). Results for *Microcystis*, which produces microcystin, had a similar pattern with the maximum *Microcystis* microcystin toxin genes (*mcyE*) occurring the week prior to the general genes (August 10 versus August 16; Fig. 3b). This has important implications for public health, because warnings are of little use when they come after the peak in toxin production.

Anatoxin-a toxin genes (*anaC*) and saxitoxin toxin genes (*sxtA*; Fig. 3d) peaked after general *Dolichospermum* (*mcyE*) genes and *Microcystis* (*mcyE*) genes (August 10, Fig. 3a,b) and at about the same time as general *Planktothrix* (*mcyE*) genes (Fig. 3c). However, the toxin gene counts by qPCR (Fig. 3d) merely indicate the potential for toxin production and therefore toxin concentrations were determined to understand when toxins are produced. Maximum saxitoxin concentrations occurred on August 23, 2016 (Fig. 2), whereas the maximum microcystin concentration occurred a week later (August 30, 2016). The timing of toxin release has important implications:

1. If water resource managers only sample for microcystin, a common practice in this region, they might miss the presence of the other toxins. For example, maximum saxitoxin concentration in Sullivan Bay occurred a week earlier than microcystin.
2. Early peaks in microcystin toxin genes (*mcyE*) may be an indication of future toxin production, when conditions (perhaps higher temperatures in combination with other environmental factors) trigger the production.

The traits of certain cyanobacteria that allow them a competitive advantage over other algae may play a role in the succession of phytoplankton species in Kabetogama Lake. *Planktothrix* can withstand the continuous mixing that is common in shallow environments like Kabetogama Lake. The lakes at VOYA are quite large, with Kabetogama Lake covering over 10,000 hectares. Therefore, wind can have a substantial mixing effect on these lakes, altering conditions such as light penetration, which can lead to fluctuating irradiance and this in turn can lead to the constant change in the dominance of different cyanobacteria. In addition, the tea-stained color of the lakes in this region^14^ may reduce light penetration, and cyanobacteria have lower light requirements than green algae^43^. With 52% of 2016 Secchi depth measurements below 1 m^40^ light availability may play an important role in the dominance of phytoplankton species. *Aphanizomenon*, in particular, are adapted to relatively low light conditions^5^.

Although urban and agricultural areas make up less than 1% of Kabetogama Lake’s watershed^44^, and typical urban and agricultural nutrient sources are absent, the nutrient source may be less important than the actual nutrient concentrations in the water. Nutrient enrichment from internal loading^13^, perhaps a remnant from the logging industry^45^ or as a result of damming^46^, may play an important role in the excess nutrients and thus the recurring algal blooms in Kabetogama Lake. Cyanobacterial blooms can be associated with poorly flushed water^34^ and research on sediment cores from Kabetogama Lake indicated that trophic state and cyanobacteria species that thrive at higher phosphorus concentrations increased since dams were constructed^47^. Total phosphorus concentrations at Kabetogama Lake during this study were between 0.028 and 0.074 milligrams per liter^40^. However, similar land use and environmental setting has not led to excess nutrients at other park lakes^14^ and, therefore, a combination of shallow depths, continual mixing, and high nutrient drainage from wetlands upstream from Kabetogama Lake^14^ in combination may lead to conditions suitable for certain cyanobacterial taxa that can access pools of phosphorus in bottom sediments^48^. Likely, a complex interplay between atmospheric conditions, hydrology, water chemistry, and the aquatic community affects temporal phytoplankton variability in Kabetogama Lake and the formation of cyanobacterial blooms.

As with any study, there are limitations on the interpretation of the data. The ELISA technique, although relatively easy and inexpensive to use, is not as rigorous as liquid chromatography/mass spectrophotometry, and data interpretation can be difficult^49^. Gene counts, at the genus specific or non-specific toxin level, may not be related one to one for the organisms that contain those genes. Some research has shown that *mcyE* gene abundance correlated to microcystin production in only a few cases^50^, and correlation between anatoxin-a or saxitoxin and *mcyE* gene abundance is not expected. Moreover, mixtures of different species of the same genus may not have the same gene copy number to cell ratio, which adds complexity to interpreting these data. Lee *et al*.^51^ reports that discrepancies between results obtained with qPCR or other molecular methods and microscopy may be due to differences in preservation, counting, or amplification of other species. Finally, toxin production is both spatially and temporally variable^52^, and this report represents only 1 year of data.

The research presented here addressed the objectives to better identify toxin producing cyanobacteria with molecular tools (qPCR) and to better understand changes in toxin production and cyanobacterial community structure (in terms of abundance, species composition, and toxin production capability) throughout an open water season. The results have furthered the understanding of cyanobacterial blooms and toxin production in remote locations, which may lead to more informed and focused remediation strategies.

Algal blooms have been blamed on excess nutrients from both urban and agricultural sources. The uniqueness of this study is the absence of urban and agricultural sources, its remote location, and the collection of samples throughout an open water season. Previous samples at this site were collected only in late summer or early fall when algal blooms were visible.

Through DNA analysis, we discovered that toxin-forming cyanobacteria were present before visible blooms and two toxins not previously detected in this region (anatoxin-a and saxitoxin) were present, making this study an important step to assess risk for ecological systems and human health. Early sampling of phytoplankton and qPCR analysis may be used as an advance warning to help resource managers decide quickly whether, when, and where to apply more rigorous or costly analytical methods.

## Materials and Methods

The data presented in this paper were part of a 2-year study (2016–2017); however, this paper covers only 2016 when phytoplankton samples were collected. Twenty-one samples were collected at the Sullivan Bay algal bloom site (482542092493701) between June 12, 2016, and September 13, 2016. All samples were collected between 8 a.m. and 3 p.m. Central Daylight Time. The exact location was sampled, whether or not a bloom was present at the time of sampling, and even when there was an obvious bloom only meters away. In general, samples were collected weekly, until August when samples were collected twice per week on subsequent days. The 21 samples were analyzed for cyanotoxins and cyanobacteria. The weekly samples (12 samples) were analyzed for phytoplankton abundance and community structure. Sampling and field measurements were made according to field methods established during previous studies in VOYA^15,24^ and generally followed standard USGS techniques for lake sampling^53^.

### Field collections and processing

Phytoplankton, cyanotoxin, and cyanobacterial gene samples were collected from just beneath the water surface with dip sampling procedures^49^. A 250-mL wide-mouthed sampling bottle was used for the phytoplankton, and a sterile 1-L polyethylene bottle was used for cyanotoxins and cyanobacterial gene samples.

Water samples were processed in a lakeside field laboratory where they were filtered and(or) preserved according to USGS methods^49,54^. Samples for phytoplankton abundance and community structure were preserved with a 9:1 Lugol’s iodine:acetic acid solution and stored in a refrigerator until batch shipment to BSA Environmental Services, Inc. (Beachwood, OH).

An aliquot of water for cyanotoxin (ELISA) analyses was extracted from the 1-L bottle, put into a 125-mL bottle, and stored in a freezer. Water for gene analyses was extracted from the 1-L bottle and filtered onto a 0.4-µm pore-size Nucleopore polycarbonate filter. The volume filtered depended on the clarity of the water, but typically sample volumes ranged from 25–100 mL. Each filter was placed into a 2-mL screw-capped vial with 0.3 grams of acid-washed beads and frozen until batch shipment. Filters were shipped to the USGS Ohio Water Microbiology Laboratory (Columbus, OH) on dry ice in batches for analyses.

### Phytoplankton analyses

Phytoplankton abundance and community structure were analyzed by microscopy for taxonomic identification and enumeration by BSA Environmental Services, Inc. (Beachwood, OH). Phytoplankton were enumerated to the lowest possible taxonomic level using membrane-filtered slides^55^. A minimum of 400 natural units (colonies, filaments, and unicells) was counted from each sample in accordance with Lund *et al*.^49,56^. Counting 400 natural units provided accuracy within 90-percent confidence limits.

### Cyanotoxin analysis

Cyanotoxins (anatoxin-a, microcystin, and saxitoxin) were analyzed in one batch per toxin at the end of the season at the USGS Ohio Water Microbiology Laboratory (Columbus, OH). The samples were lysed by three sequential-freeze/thaw cycles followed by syringe filtration with a 0.7-µm glass-fiber syringe filter and analyzed by ELISA kits acquired from Abraxis, LLC (Warminster, PA) for microcystins/nodularins^57^, anatoxin-a, and saxitoxins^19^. The minimum reporting levels (MRL) are 0.3 μg/L as anatoxin-a equivalents, 0.3 µg/L as total microcystin and nodularin expressed as microcystin-LR equivalents (called microcystin throughout this paper), and 0.02 μg/L as saxitoxin equivalents. Samples exceeding the highest calibration standard were diluted.

### Cyanobacterial gene analysis

Samples were extracted and analyzed for cyanobacterial genes at the USGS Ohio Water Microbiology Laboratory (Columbus, OH) by use of qPCR according to procedures in Stelzer *et al*.^58^, summarized below. Molecular assays for cyanobacterial genes were analyzed to enumerate the following:

- General cyanobacteria^59^
- Genus-specific assays for *Dolichospermum*, *Microcystis*, and *Planktothrix*^59–61^
- Genus-specific microcystin (*mcyE*) toxin gene assays for *Dolichospermum*, *Microcystis*, and *Planktothrix*^18,21^
- Saxitoxin (*sxtA*) toxin gene^62^
- Anatoxin-a (*anaC*) toxin gene^22^

### DNA extraction and qPCR analysis

Samples were extracted^58^ by use of a DNA-EZ extraction kit (GeneRite, North Brunswick, NJ) according to manufacturer’s instructions, except that no prefilter was used and the final elution volume was 150 microliters (µL). An extraction blank was included with each batch of sample extractions.

From each sample extract, 5 µL was analyzed by qPCR in duplicate for each assay described above by using primer and probe sets as well as qPCR performance conditions detailed in the lysed references for each assay. All assays were performed on either an Applied Biosystems 7500™ or a StepOnePlus™ (Life Technologies, Carlsbad, CA) thermal cycler. TaqMan™ Universal PCR Master Mix (Life Technologies, Carlsbad, CA) was used if the assay included a probe, whereas SYBR™ Green PCR Master Mix (Life Technologies, Carlsbad, CA) was used if the assay did not include a probe. If the sample results were considered inhibited, the extracts were diluted and reanalyzed^58^. Results from diluted reanalyzed samples were used to compute final concentrations.

### Standard curves and quantifying cyanobacterial toxin genes

Standards were included in duplicate with each qPCR run to construct a seven-point standard curve. Plasmid standards for each assay were used to establish standard curves for quantification. Before qPCR analysis, plasmid containing *Escherichia coli* was grown overnight and plasmids were extracted and purified from the *E. coli* cells using the QuickLyse Miniprep Kit (Qiagen, Valencia, CA). The copy number of each target was calculated using the DNA concentration measured by the Qubit™ dsDNA High Sensitivity Assay (Life Technologies, Carlsbad, CA) and the molecular weight of the plasmid. Sample results were reported as copies per 100 milliliters (copies/100 mL). Standard curve characteristics for this study are listed in Table 4.

**Table 4:** Standard curve characteristics for quantitative polymerase chain reaction (qPCR) analyses performed by the U.S. Geological Survey Ohio Water Microbiology Laboratory during this study of Kabetogama Lake Sullivan Bay, Northwest, near Ash River (U.S. Geological Survey Site No. 482542092493701), Voyageurs National Park, MN, USA, 2016.

qPCR assay	Dynamic range	Amplification efficiency (percent)	R^2^ value	LoD	LoQ
general cyanobacteria	66.0–6.60E + 07	95–97	0.995–0.996	26	72
general *Microcystis*	9.44–9.44E + 06	91–96	0.994–0.997	27	55
general *Planktothrix*	16.1–1.61E + 07	99–100	0.998–0.999	18	34
general *Dolichospermum*	24.6–2.46E + 07	98–100	0.992–0.995	230	550
*Microcystis* microcystin toxin gene (*mcyE*)	11.4–1.14E + 07	93–98	0.997	13	39
*Planktothrix* microcystin toxin gene (*mcyE*)	28.4–2.84E + 07	94–95	0.993–0.998	51	110
*Dolichospermum* microcystin toxin gene (*mcyE*)	19.2–1.92E + 07	95–96	0.997–0.998	46	120
saxitoxin toxin gene (*sxtA*)	30.8–3.08E + 07	81–84	0.995–0.998	14	45
anatoxin-a toxin gene (*anaC*)	74.5–7.45E + 07	83–84	0.999	32	60

[qPCR, quantitative polymerase chain reaction; dynamic range, limit of detection, and limit of quantification are reported in copies per reaction; R^2^, coefficient of determination; LoD, limit of detection; LoQ, limit of quantification].

The limit of detection (LoD) and limit of quantification (LoQ) were calculated for each assay^63^, in addition to the coefficient of determination (R^2^). The LoD and LoQ values were initially cycle threshold values converted to and reported as copies per qPCR reaction by use of an assay-specific standard curve. Sample results lower than the LoQ but above the LoD are reported as estimated values. Because original sample volumes and dilutions to overcome inhibition were often different for each sample or set of samples, the LoD was applied on a sample-by-sample basis to determine sample reporting limits. The sample reporting limits are the “less-than values” for each sample and assay and are also reported as copies/100 mL.

### Quality assurance and quality control

Quality-control samples, consisting of replicates, blanks, and spikes were analyzed to document the variability associated with sample collection and laboratory procedures. Two field quality-assurance samples (one replicate and one blank) were collected and analyzed in 2016, which is approximately 16% of the environmental samples. Additional field quality samples were analyzed at other field sites for the larger study effort, and median relative percent difference for all replicate samples was between 0 and 10 percent^28^.

Effectiveness of equipment cleaning and sample processing was assessed by laboratory analysis of field blanks. Laboratory blank water was processed in the field with the same collection bottles, filtering devices, and methods as for native water samples. For cyanobacterial gene samples, one additional filter blank using sterile water was filtered each day samples were processed. If detections are found in the blank samples, the limit of blanks is calculated and used in determining the detection limit of an assay. This is defined as the 95th percentile of all cycle threshold values for the blanks for a specific assay.

At the USGS Ohio Water Microbiology Laboratory, laboratory replicates were analyzed with each batch and considered acceptable at a coefficient of variance (% CV) equivalent to +/− 20% of average or expected value or less. If laboratory replicate samples did not meet this criterion, then the entire plate was reanalyzed. More variation may be expected with field replicates due to difficulty with homogenizing intact cyanobacterial cells when splitting samples; therefore, field replicate samples were not reanalyzed if percent relative standard deviation (RSD) exceeded +/− 20%, but rather accepted as inherent variability due to temporal or spatial variation.

### Ethical approval

This article does not contain any studies with human participants performed by any of the authors.

## Data Availability

All data generated or analyzed during this study are publicly available and included in a USGS ScienceBase Catalog data release^28^ or through the USGS National Water Information System^40^ by accessing site number: 482542092493701.
